# Supplementary material for: White-light activatable organic NIR-II luminescence nanomaterials for imaging-guided surgery
Source: Nat Commun. 2024 Jul 11;15:5832. doi: 10.1038/s41467-024-50202-6 (PMC11239823; doi:10.1038/s41467-024-50202-6)
Supplement: Supplementary file 1 — Supplementary Information [file 41467_2024_50202_MOESM1_ESM.pdf]

Supplementary Information

White-light activatable organic NIR-II luminescence nanomaterials for imaging-guided surgery

Chunbin Li[1,†], Jian Du[2,†], Guoyu Jiang[1,†], Jianye Gong[1], Yue Zhang[1], Mengfan Yao[1], Jianguo Wang[1*], Limin Wu[1,3*], Ben Zhong Tang[4]

[1]College of Chemistry and Chemical Engineering, College of Energy Material and Chemistry, Inner Mongolia Key Laboratory of Fine Organic Synthesis, Inner Mongolia University, Hohhot 010021, China.

[2]Department of Urology, The First Affiliated Hospital of Shandong First Medical University, Jinan, Shandong 250000, China.

[3]Department of Materials Science and State Key Laboratory of Molecular Engineering of Polymers, Fudan University, Shanghai 200433, China.

[4]School of Science and Engineering, Shenzhen Institute of Aggregate Science and Technology, The Chinese University of Hong Kong, Shenzhen (CUHK-Shenzhen), Guangdong 518172, China.

†These authors contributed equally to this work

*Corresponding author. Email: wangjg@iccas.ac.cn (J. W.), wlm@imu.edu.cn (L. W.)

Experimental Section/Methods

Materials and Instruments. All the chemicals and reagents were purchased from Adamas-beta®, Sigma-Aldrich without additional treatment before use. 12,13-bis(2-ethylhexyl)-3,9-diundecyl-12,13-dihydro-[1,2,5]thiadiazolo[3,4-e]thieno[2",3":4',5']thieno[2',3':4,5]pyrrolo[3,2-g]thieno[2',3':4,5]thieno[3,2-b]indole-2,10-dicarbaldehyde, 2-(4-oxocyclopenta[c]thiophen-6-ylidene) propanedinitrile were purchased from Derthon Optoelectronic Materials Science Technology Co., Ltd. Thiazolyl blue tetrazolium bromide (MTT) was purchased from Beyotime biotechnology Co., Ltd. Indocyanine green (ICG), 1-butyl-2-[2-[3-[2-(1-butyl-6-chlorobenz[cd]indol-2(1H)-ylidene)ethylidene]-2-chloro-1-cyclohexen-1-yl]ethenyl]-6-chlorobenz[cd]indolium tetrafluoroborate (IR1048) was purchased from Sigma-Aldrich. IR26 dye was purchased from Xi'an ruixi Biological Technology Co. Ltd.

Structural characterization. The organic compounds synthesized were characterized for their structures and purities using ^{1}H and ^{13}C NMR spectroscopy with Bruker ARX 600 and ARX 500 spectrometers, using chloroform-*d* as the solvent and tetramethylsilane (TMS) as a reference. High-resolution mass spectra (HRMS) were obtained using a GCT Premier CAB 048 mass spectrometer to determine exact molecular weights. Molecular configurations were elucidated through single crystal X-ray diffractometer using Bruker smart Apex 2 and Bruker D8 Venture. **Nanoparticle characterization:** Particle size and morphology were examined using a Hitachi HT 7800 transmission electron microscope, with size distribution analyzed *via* dynamic light scattering (DLS) using an Omni NanoBrook device. **Photophysical characterization:** The UV-*vis*-NIR and photoluminescence (PL) spectra were recorded using a Shimadzu UV-2600i spectrophotometer and an FS5 Spectrofluorometer, respectively. Temperature curves were monitored using an FLIR E8-XT camera (FLIR System). **Theoretical calculations:** Weak interaction analysis from single crystal structure were performed using Multiwfn, and corresponding structure and IGM isosurfaces were generated using the VMD program. **Cellular experiment:** The absorbance of each sample was measured using a microplate reader (BioTek) for MTT assay. **NIR-II imaging:** A Series III 900/1700 In Vivo Imaging System (Suzhou NIR-Optics Co., Ltd., China) was used for image acquisition. In addition, excitation sources for photophysical tests, cellular, and *in vivo* experiments included laparoscopic-light (XD-303-80W, LED cold light source) and an 808 nm laser (CNI laser, MDL-XD-808-5W).

Preparation of nanoparticles. The NPs were synthesized using the nanoprecipitation technique. Initially, 1 mg of the compound and 5 mg of DSPE-mPEG$_{2000}$ were dissolved in 1 mL of THF to create a uniform solution. This mixture was then injected into 9 mL of double distilled water and subjected to ultrasonication for 5 min. Subsequent to this, the solution was transferred into a dialysis bag with a

molecular weight cutoff of 3600 and dialyzed against deionized water for 24 h. Finally, the NPs were concentrated and subsequently calibrated to determine their concentration.

Fluorescence quantum yield. The QY of a sample was determined using the NIR-II fluorescent IR26 dye (QY = 0.5%) as a reference[1,2]. The fluorescence emission spectrum of the IR26 dye in 1,2-dichloroethane (DCE) solution was measured under 808 nm laser excitation, with absorbance values ranging from 0.02 to 0.10. Subsequently, the absorption and emission of the nanoparticles in water or fetal bovine serum (FBS) were measured using an Edinburgh FS5 fluorescence spectrophotometer. The integrated photoluminescence intensity (wavelength range: 850-1400 nm and 1000-1400 nm) was recorded against the absorbance at the excitation wavelength of 808 nm and fitted to a linear function. This was used to calculate the QY of the sample, based on equation (a):

$$QY_{\mathrm{sample}} = QY_{\mathrm{ref}} \times \frac{slope_{\mathrm{sample}}}{slope_{\mathrm{ref}}} \times (\frac{n_{\mathrm{sample}}}{n_{\mathrm{ref}}})^2 \qquad \text{(a)}$$

where $slope_{\mathrm{sample}}$ and $slope_{\mathrm{ref}}$ are the slope of sample in water and IR26 in DCE, respectively, which are obtained after linear fitting curves within certain ranges. The n_{sample} is the refractive index of H_2O or FBS, and the n_{ref} is the refractive index of DCE.

Photothermal properties of Y6CT-NPs. The white-light power density was calibrated with a spectro-densitometer to ensure accuracy. The temperature curves of Y6CT-NPs (200 μL) in water were recorded at various concentrations (0, 20, 50, 100 μM) upon white-light exposure (20 mW cm^{-2}) for 6 min.

Crystal growth. Y6CT (3 mg) was placed in a 5 mL glass bottle containing CH_2Br_2 (1 mL) and was sealed within a larger 20 mL glass bottle filled with methanol (~ 5 mL). The container was undisturbed at room temperature until crystal growth was observed (5 ~ 10 days). The Crystallographic and structural refinement data of Y6CT are presented in Supplementary Table 2.

Theory calculations. IGM analysis of weak interaction based on single crystal structure was conducted by using Multiwfn 3.8(dev). The corresponding structure and IGM isosurfaces were generated using VMD 1.9.3 program.

Cell viability. Human normal liver cell line LO2 (ATCC® CRL-12461) and mouse embryonic fibroblast cell line NIH-3T3 (ATCC® CRL-1658) were cultured in DMEM supplemented with 10% FBS, 100 mg mL^{-1} penicillin, and 100 mg mL^{-1} streptomycin at 37 °C in a humidified incubator with 5% CO_2. After reaching confluence, 100 μL of cell suspension (~ 5000 cells) was added to each well of a 96-well plate and pre-incubated for 24 h. Subsequently, various concentrations of Y6CT-NPs (0, 20, 40, 60, 80, 100 μM) were added to the plate and incubated for 20 h. The plate was then exposed to laparoscopic LED

light (50 mW cm^{-2}) for 10 min and cells without any treatment as a dark group. After 4 h of normal nurturing at 37 °C, the serum-containing media was replaced with serum-free media and MTT reagent in cell cultures for 4 h, followed by the addition of 100 µL of DMSO to dissolve the formazan crystals. Absorbance was measured at 490 nm using an ELISA Plate Reader (Biotek). The cells incubated with a culture medium was used as a control. All experiments were conducted in triplicate. Cell viability was calculated using the following formula:

$$\text{Cell viability (\%)} = \frac{A_{\text{Sample}} - A_0}{A_{\text{Control}} - A_0} \times 100\% \quad \text{(b)}$$

where A_{Sample} and A_{Control} are the absorbance of cells treated and untreated sample, respectively. A_0 is the absorbance without cells.

Hemocompatibility test. Whole blood was collected from healthy mice, centrifuged at 500 g for 10 min to separate the serum and blood cells. The blood cells were then washed thrice with phosphate buffer saline buffer (PBS). Subsequently, 50 µL of blood cells were treated with varying concentrations of Y6CT-NPs (1, 2, 5, 10, 20, 30, 100 µM) in polyethylene pipe containing 1 mL of PBS, and incubated for 2 h at 37 °C. PBS and 1% Triton X-100 (Triton) were used as negative control and positive control, respectively. Then, all samples were centrifuged for 10 min, and images were captured. The absorbance of the supernatant was measured at 540 nm. To correct for compound absorbance, a Y6CT-NPs in PBS solution with the same concentration was used as the blank group. Hemolysis was calculated using the following formula:

$$Hemolysis\ (\%) = \frac{A_{\text{Blood}} - A_{\text{Sample}}}{A_{\text{Triton}} - A_{\text{PBS}}} \times 100\% \quad \text{(c)}$$

where A_{Blood} and A_{Sample} represent the absorbance of blood-containing and blood-free PBS solution treated with varying concentrations of Y6CT-NPs, respectively. A_{Triton} and A_{PBS} denote the absorbance of blood-containing PBS solution treated with and without Triton X-100, respectively.

Metabolic pathway of Y6CT-NPs. BALB/c nude mice were intravenously administered Y6CT-NPs (300 µM, 100 µL), followed by euthanasia on the 1st, 3rd, 5th, and 7th days post-injection. Subsequently, the major organs (heart, liver, spleen, lung, kidney) of these mice were excised, and their fluorescent images were acquired utilizing the NIR-II fluorescence imaging system with 1000 nm long-pass (LP) filter upon activation by white-light illumination (16.5 mW cm^{-2}).

***In vivo* pharmacokinetics study.** BALB/c nude mice were given Y6CT-NPs (300 µM, 100 µL) intravenously and blood was taken from tail artery at different intervals (2/60, 4/60, 6/60, 8/60, 15/60, 30/60, 1, 2, 3, 4, 6, 8, 10 and 12 h) and stored in heparinized tubes. The fluorescence intensity of Y6CT-

NPs was determined with the aid of NIR-II fluorescence imaging instrument under white-light illumination (16.5 mW cm^{-2}) activation to create the calibration curve by plotting intensity versus concentration.

Histological study. In order to assess the *in vivo* safety of Y6CT-NPs, two groups of mice (n = 3 independent samples per group) and New Zealand rabbits (n = 3 independent samples per group) were used for the study of short-term toxicity. The imaging group of the mice were administered with 100 µL of Y6CT-NPs (300 µM) intravenously while the control group was not given any treatment. Similarly, the imaging group of rabbits were injected with 2 mL of Y6CT-NPs (300 µM) intravenously while the control group of rabbits was left untreated. All the mice and rabbits were sacrificed 4 hours after the vessels imaging. The heart, lungs, spleen, kidneys, ureters, and bladders of each animal were then collected, fixed in 4% paraformaldehyde overnight at 4 °C, and then embedded in paraffin. The histological sections were further stained with hematoxylin and eosin (H&E) and imaged under an inverted optical microscope with an objective lens of 20X.

Blood analysis and liver and kidney function tests. The imaging and control groups for both mice and New Zealand rabbits were divided, each consisting of three animals. The imaging group for mice was administered 100 µL of Y6CT-NPs (300 µM) intravenously, while the control group was not treated. Following this, 4 h post-injection, blood samples were collected and used to measure liver and kidney function, including ALT (alanine aminotransferase), AST (aspartate aminotransferase), ALB (albumin), BUN (blood urea nitrogen), CREA (creatinine), and UA (uric acid) using detection kits as per the manufacturer's instructions. Additionally, blood indexes of the animals were measured with a hemocytometer. For the New Zealand rabbits, the imaging group was given 2 mL of Y6CT-NPs (300 µM) intravenously, while the control group was not treated. Similarly, after 4 h post-injection, the same blood tests and hemocytometer analyses were conducted to evaluate liver and kidney function.

Data analysis. All statistical graphs, absorption spectrum, and fluorescent spectra were analyzed with OriginLab 2018. NMR files were analyzed with MestReNova. Mass spectrum files were analyzed with flexAnalysis. Each experiment included at least three replicates. For photostability in Fig. 1h, the statistical significance of differences between groups was performed by using an unpaired two-sided *t*-test method on the SPSS 23 software. A value of $P < 0.05$ was considered significant and were indicated with asterisks: *P < 0.05, **P < 0.01 and ***P < 0.001.

Synthesis of Y6CT.

2-(5,6-dihydro-6-oxo-4H-cyclopenta[c]thien-4-ylidene)propanedinitrile (CPT30, 200 mg, 1 mmol), 12,13-bis(2-ethylhexyl)-3,9-diundecyl-12,13-dihydro-[1,2,5]thiadiazolo[3,4e]thieno[2",3'':4',5']thieno [2',3':4,5]pyrrolo[3,2-g]thieno[2',3':4,5]thieno[3,2-b]indole-2,10-dicarbaldehyde (TPB-CHO, 513 mg,

0.5 mmol) and pyridine (0.5 mL) were fully dissolved in 20 mL trichloromethane ($CHCl_3$) under N_2 atmosphere. After stirring the mixture for 12 h at room temperature. The reaction was monitored until aldehyde compound was fully consumed (determined by TLC), then purified by silica gel chromatography with the mixed solvent of petroleum ether/dichloromethane (1:1, *v*/*v*) to give brown black solid product. ^{1}H NMR (600 MHz, Chloroform-*d*) δ 9.04 (s, 2H), 8.36 (s, 2H), 7.97 (s, 2H), 4.78 (d, J = 7.8, 4H), 3.22 (t, J = 8.1, 4H), 2.23 – 2.11 (m, 2H), 1.89 – 1.84 (m, 4H), 1.53 – 1.47 (m, 4H), 1.38 – 0.96 (m, 44H), 0.87 – 0.85 (m, 6H), 0.81 – 0.78 (m, 6H), 0.67 – 0.64 (m, 6H). ^{13}C NMR (126 MHz, Chloroform-*d*) δ 181.76, 156.76, 153.27, 147.37, 145.08, 142.60, 142.33, 137.93, 136.26, 135.80, 133.48, 133.27, 129.93, 127.40, 127.06, 125.24, 115.34, 115.05, 113.60, 66.51, 55.64, 40.28, 31.93, 31.18, 29.83, 29.66, 29.63, 29.59, 29.53, 29.51, 29.35, 27.55, 27.54, 23.24, 22.83, 22.70, 14.14, 13.73, 10.35. HRMS: m/z: [M+CH_3OH-H]$^+$ calcd for $C_{79}H_{89}N_8O_3S_7$: 1421.51026; found: 1421.52405.

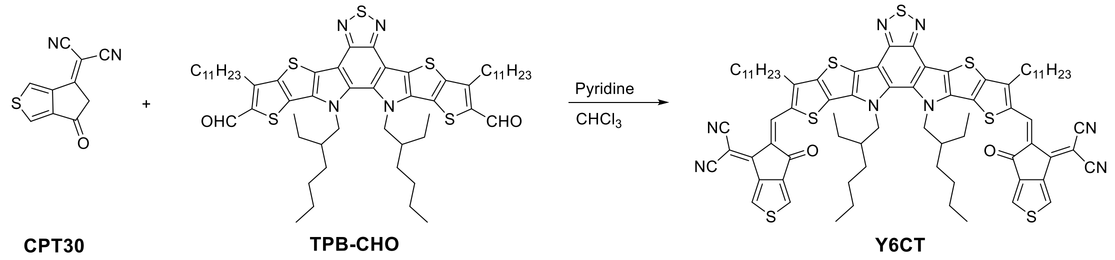

Supplementary Fig. 1. Synthetic routes to Y6CT.

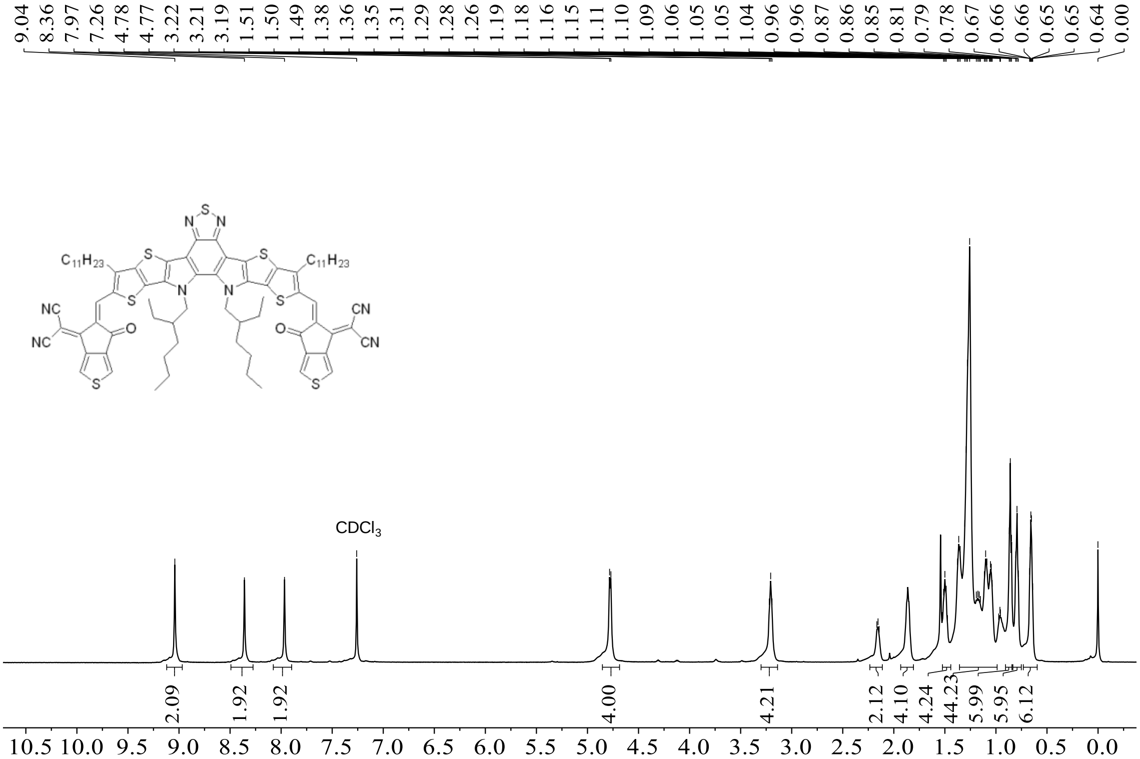

Supplementary Fig. 2. ^{1}H NMR spectrum of Y6CT in chloroform-*d*.

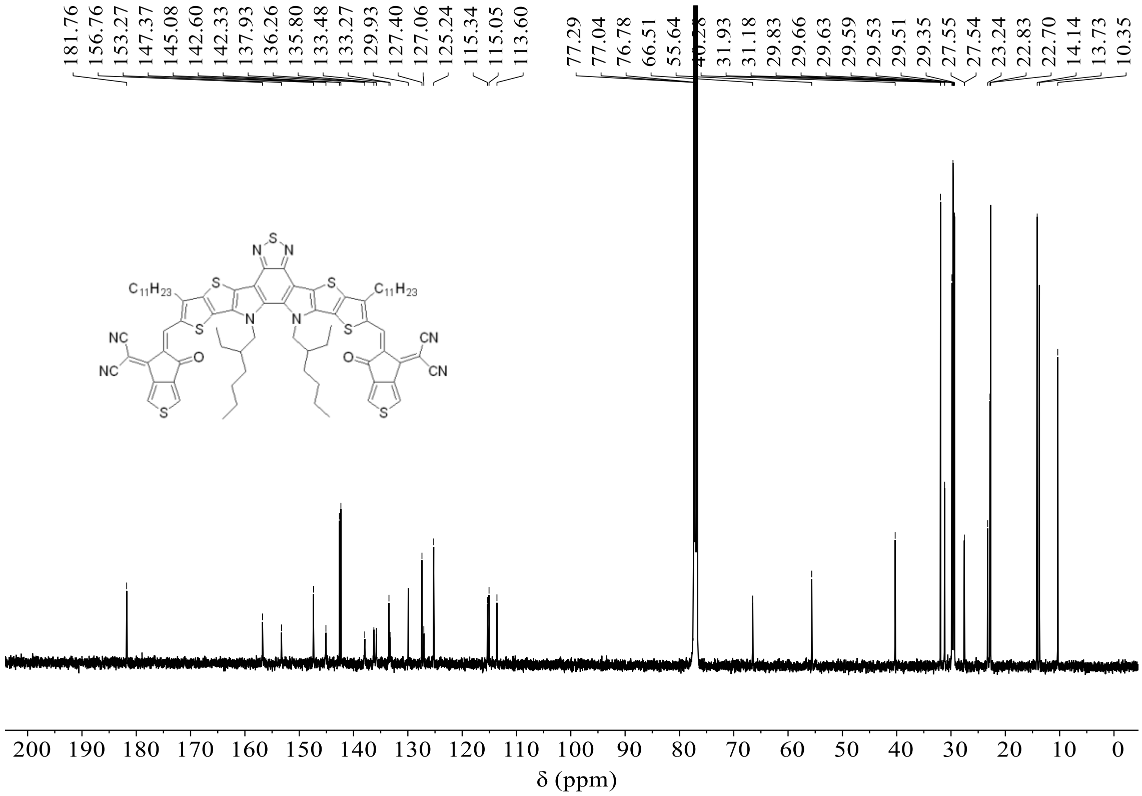

Supplementary Fig. 3. ^{13}C NMR spectrum of Y6CT in chloroform-*d*.

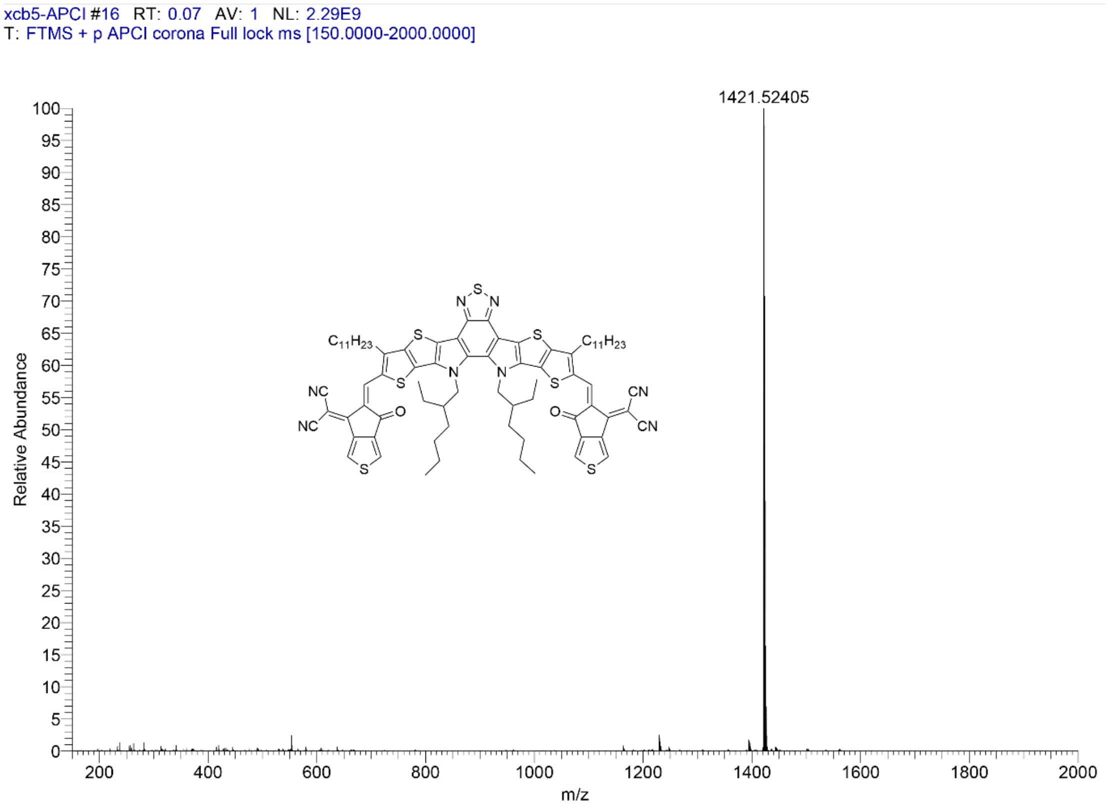

Supplementary Fig. 4. HR-MS spectrum of Y6CT.

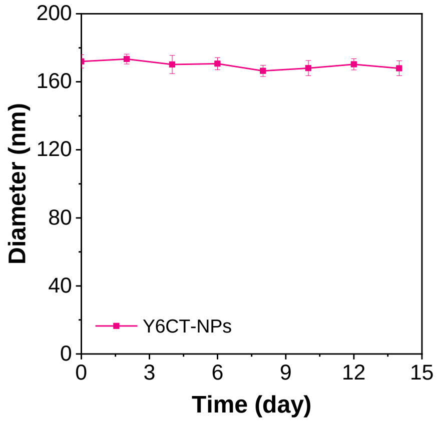

Supplementary Fig. 5. The diameter changes of Y6CT-NPs in PBS within 14 days. Data were presented as mean ± SD derived from n = 3 independent samples per group.

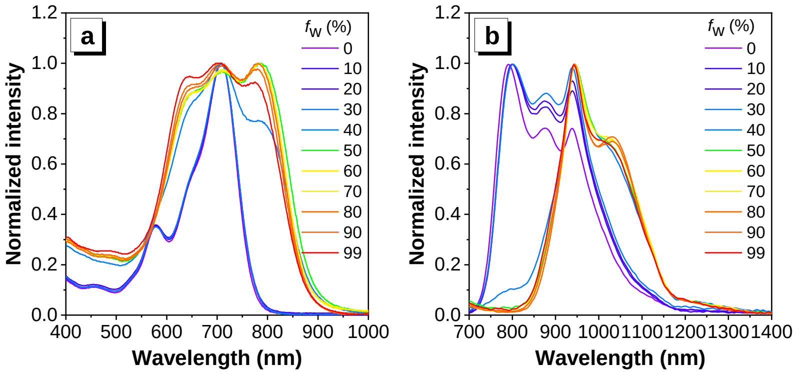

Supplementary Fig. 6. a-b The normalized UV-*vis*-NIR spectra (**a**) and PL spectra (**b**) of Y6CT (10 μM) in a mixture of THF and water with varying water fractions (f_w).

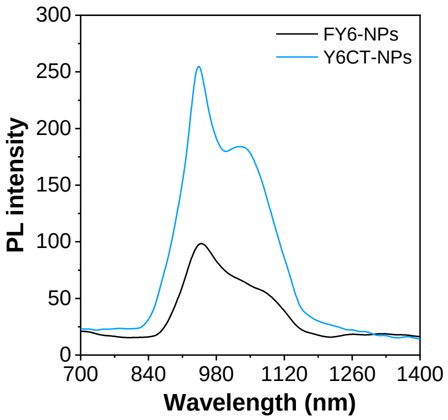

Supplementary Fig. 7. Fluorescence spectra of FY6-NPs and Y6CT-NPs (20 μM, E_x = Laparoscopic-light, 643 nm LP filter).

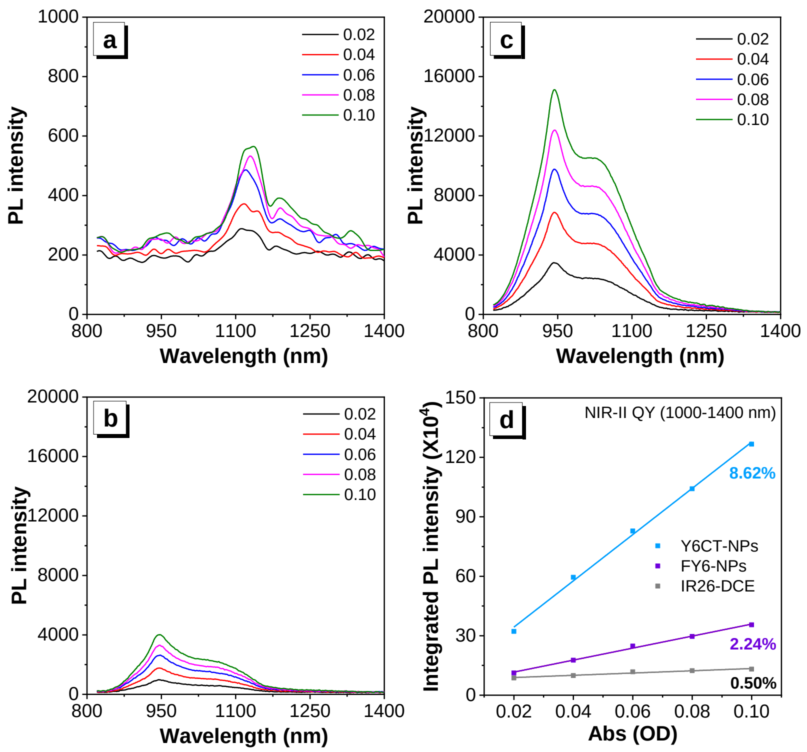

Supplementary Fig. 8. a-c The fluorescence spectra of IR26 in DCE (**a**), FY6-NPs (**b**) and Y6CT-NPs in deionized water (**c**) with different optical densities (ODs) under 808 nm laser excitation. **d** The linear fitting of the integrated PL intensity in the range of 1000-1400 nm *vs.* the absorbance values of Y6CT-NPs, FY6-NPs and IR26.

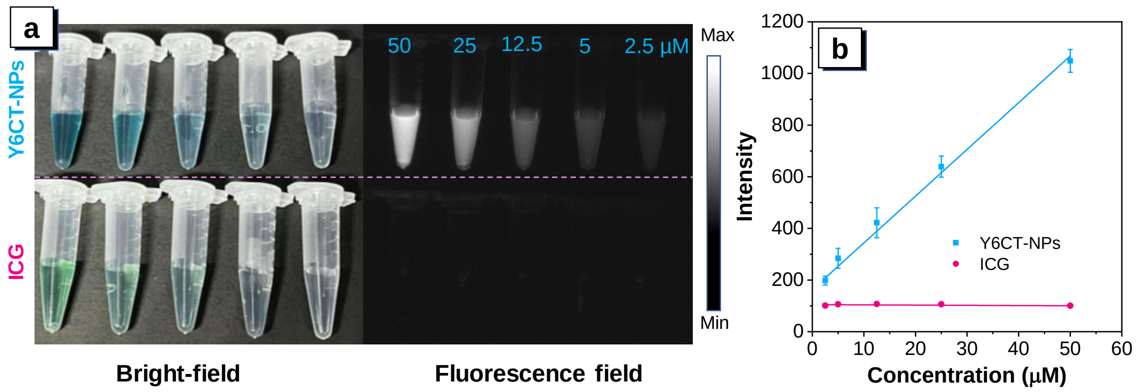

Supplementary Fig. 9. a-b The photographs of Y6CT-NPs and ICG with different concentration (2.5, 5, 12.5, 25, 50 μM) under daylight and NIR-II fluorescence images upon white-light illumination (16.5 mW cm^{-2}) activation (**a**), and corresponding average fluorescence intensity of Y6CT-NPs and ICG in water at different concentration (**b**). Data were presented as mean ± SD derived from n = 3 independent samples per group.

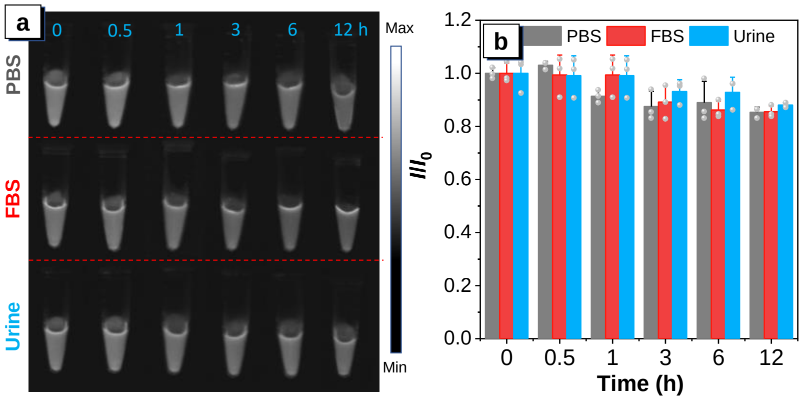

Supplementary Fig. 10. a-b The NIR-II fluorescent images (**a**) and corresponding fluorescence intensity ratio (I/I_0) of Y6CT-NPs (20 μM, 200 μL) (**b**) with varying times at 37 °C. *I* represents the PL intensity of the samples at different time, while I_0 indicates the initial intensity at 0 min. E_x = White-light illumination. Data were presented as mean ± SD derived from n = 3 independent samples per group.

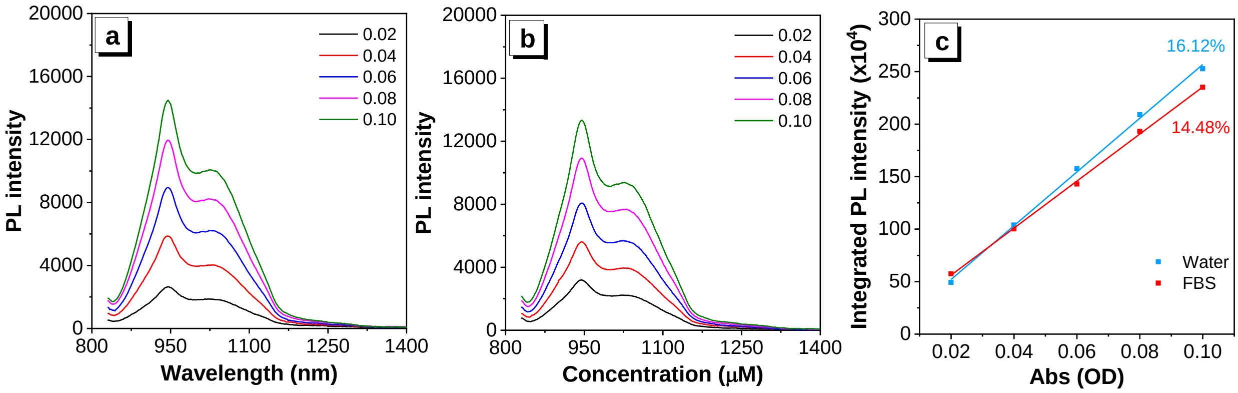

Supplementary Fig. 11. a-b The fluorescence spectra of Y6CT-NPs in deionized water (**a**) and fetal bovine serum (FBS) with different ODs under 808 nm laser excitation (**b**). **c** The linear fitting of the integrated PL intensity in the range of 850-1400 nm *vs*. the absorbance values of Y6CT-NPs in water and FBS.

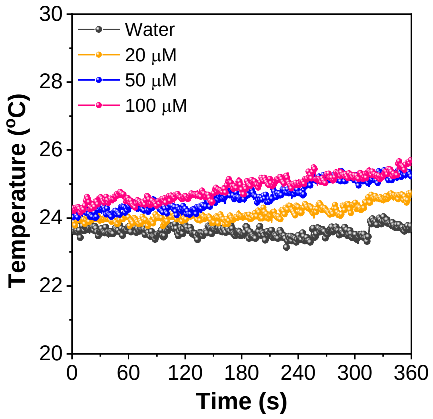

Supplementary Fig. 12. Photothermal curves of Y6CT-NPs with different concentrations under laparoscopic-light excitation (20 mW cm^{-2}).

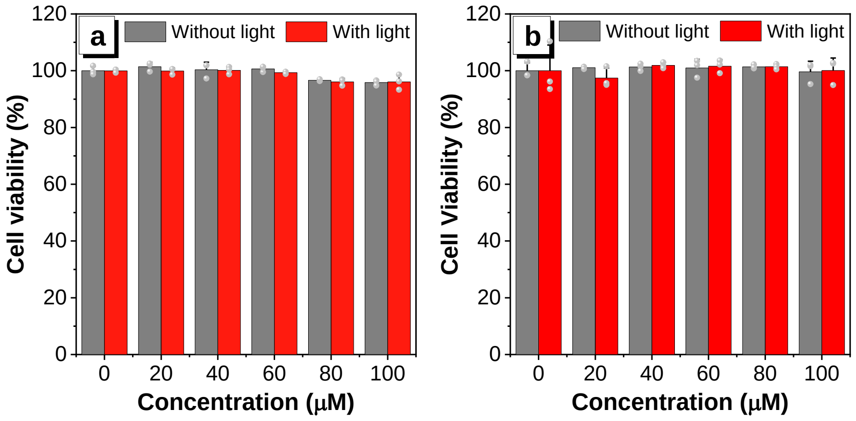

Supplementary Fig. 13. **a-b** Viability of LO2 (**a**) and NIH-3T3 cells (**b**) incubated with Y6CT-NPs at varying concentrations in the dark or with laparoscopic-light activation for 10 min (50 mW cm^{-2}). Data were presented as mean ± SD derived from n = 3 independent samples per group.

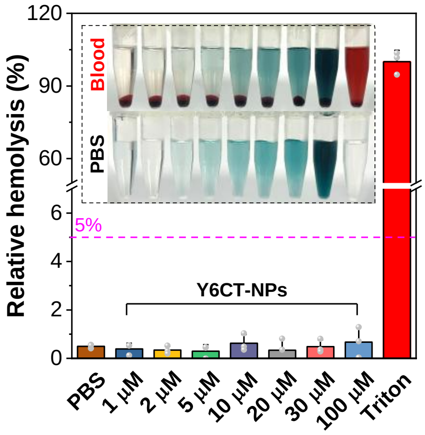

Supplementary Fig. 14. The hemocompatibility of Y6CT-NPs. Hemolytic percentage of red blood cells after treatment with Y6CT-NPs at various concentrations ranging from 1 to 100 μM after 2 h incubation. PBS and 1% Triton X-100 (Triton) were used as negative control and positive control, respectively. Data were presented as mean ± SD derived from n = 3 independent samples per group.

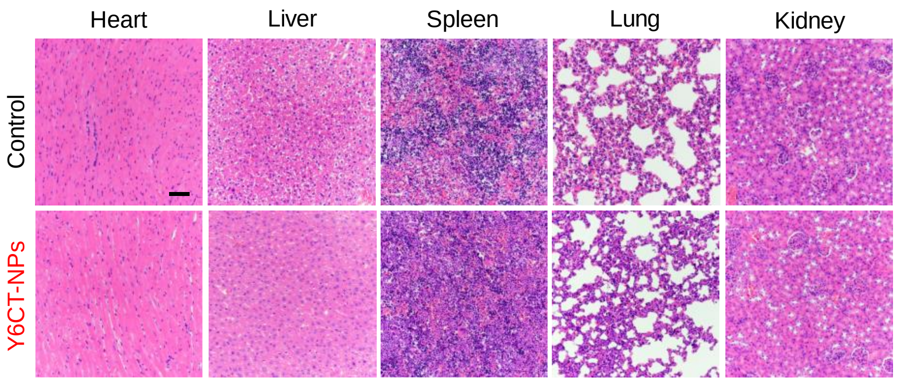

Supplementary Fig. 15. Histological H&E staining of major organs (heart, liver, spleen, lung, kidney) of mice in different groups. Scale bar: 100 μm.

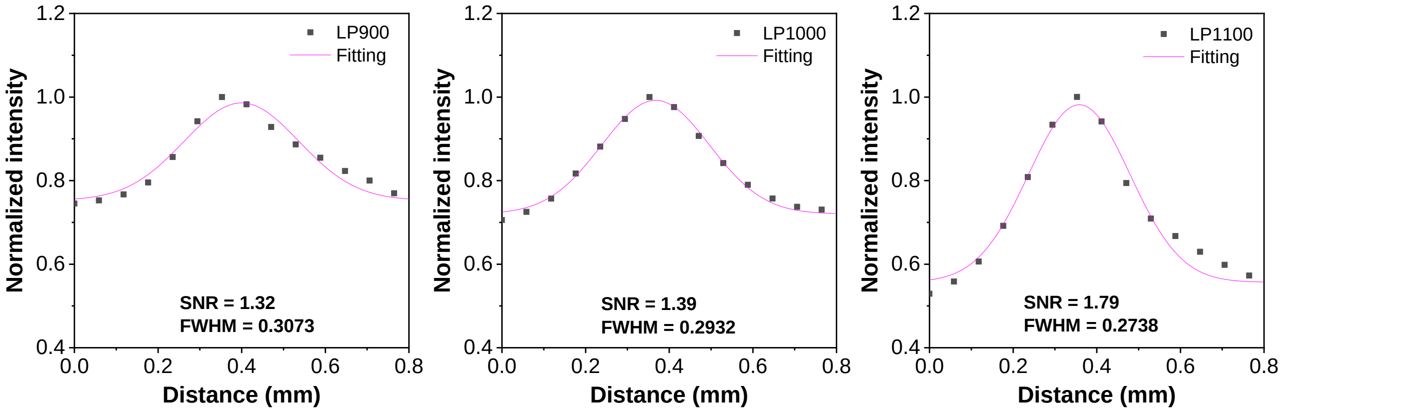

Supplementary Fig. 16. Cross-sectional intensity profiles (dark dots) and Gaussian fit (pink lines) along the pink line of abdominal vasculature in Fig. 3a with different LP filters.

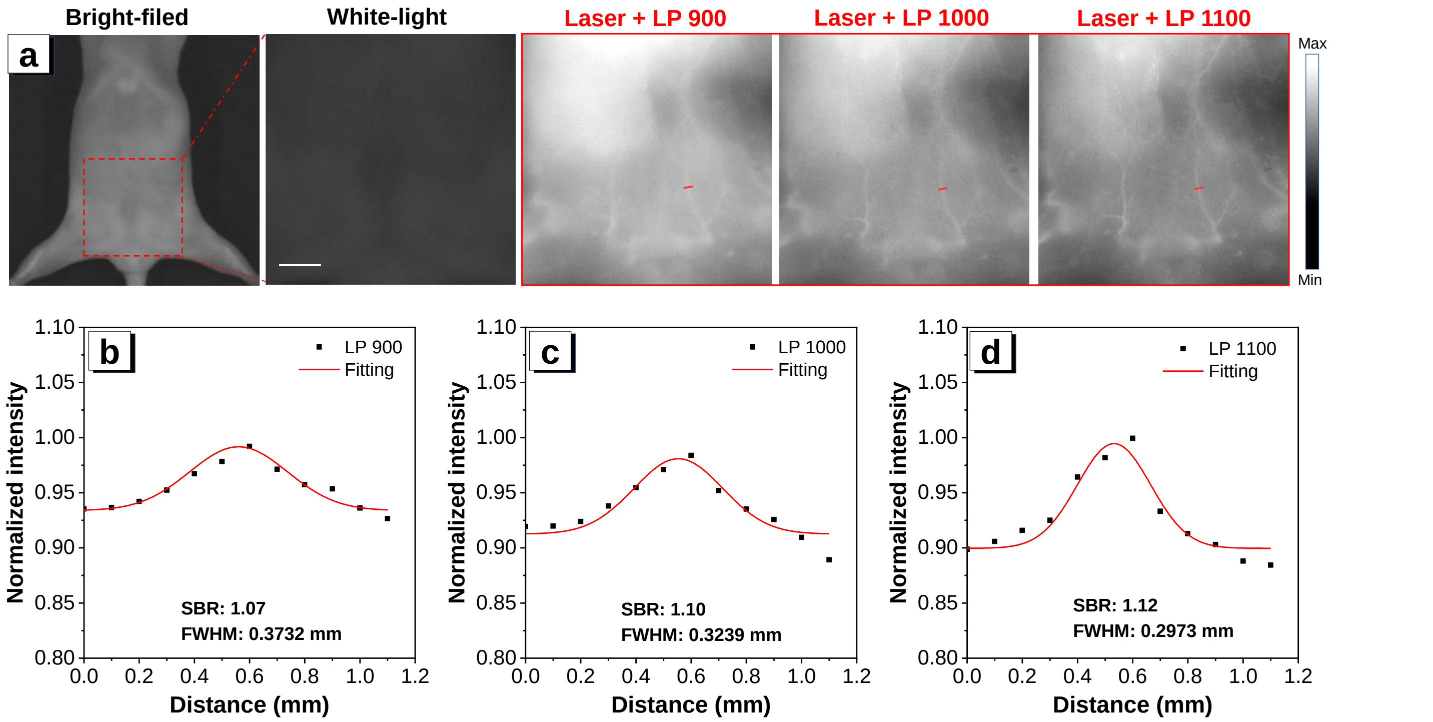

Supplementary Fig. 17. a NIR-II fluorescence imaging of blood vessels of BALB/c mice in supine positions after *i.v.* injection of 100 μL ICG (300 μM, 100 μL) under white-light irradiation and 808 nm laser at the same optical power density (16.5 mW cm^{-2}). Repeated for three times in independent experiments. **b-d** Cross-sectional intensity profiles (dark dots) and Gaussian fit (red lines) along the pink line of abdominal vasculature with 900 nm (**b**), 1000 nm (**c**), 1100 nm (**d**) LP filters. Scale bar: 5 mm.

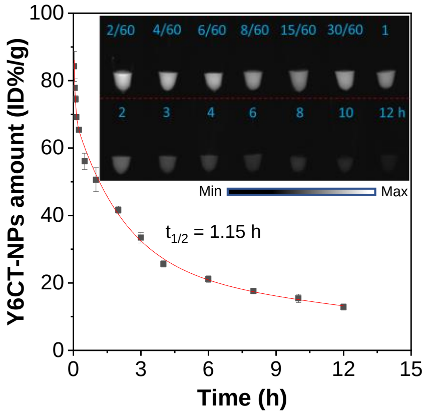

Supplementary Fig. 18. Pharmacokinetics profile and fitted curve of Y6CT-NPs in blood at different time after intravenous injection. Inset: Fluorescent images of blood in mice at different time after intravenous injection of Y6CT-NPs (300 μM, 100 μL). E_x = White-light illumination (16.5 mW cm^{-2}). Data were presented as mean ± SD derived from n = 3 independent samples.

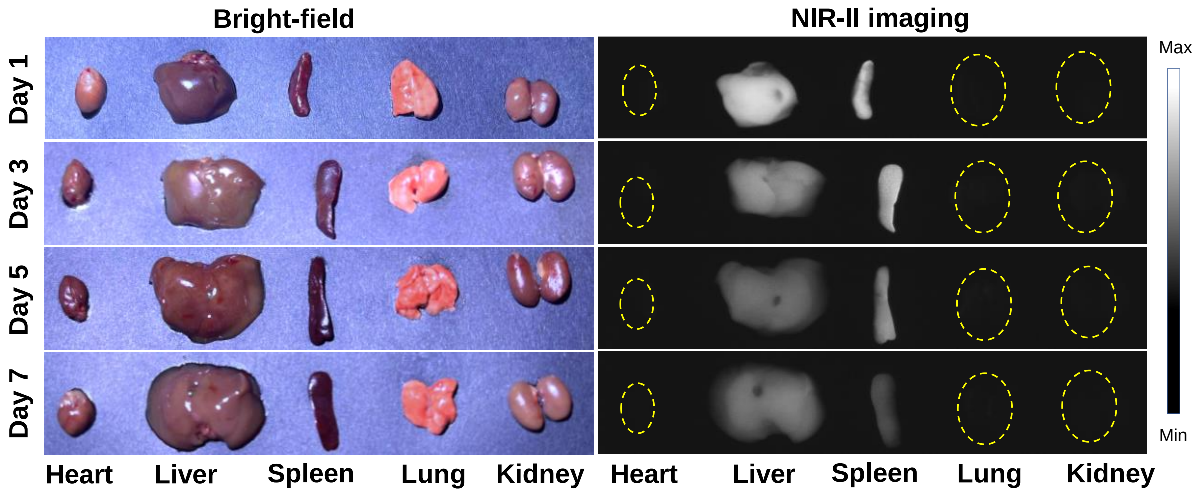

Supplementary Fig. 19. The photographs and NIR-II imaging of major organs (heart, liver, spleen, lung, kidney) at 1st, 3rd, 5th, and 7th days post-injection of Y6CT-NPs (300 μM, 100 μL). E_x = White-light illumination (16.5 mW cm^{-2}).

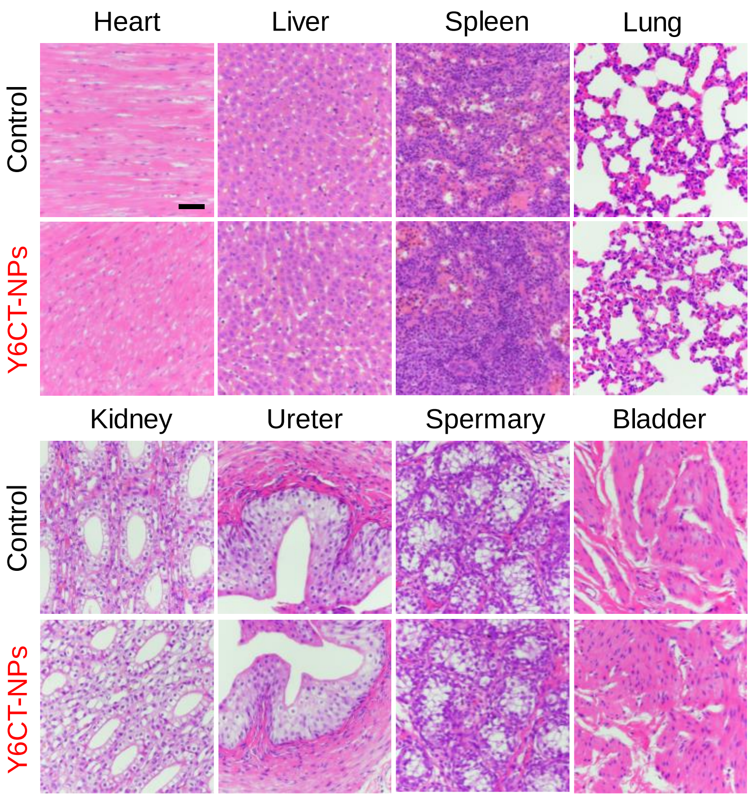

Supplementary Fig. 20. Histological H&E staining of major organs (heart, liver, spleen, lung, kidney, ureter, spermary, bladder) of rabbit in different groups. Scale bar: 100 μm.

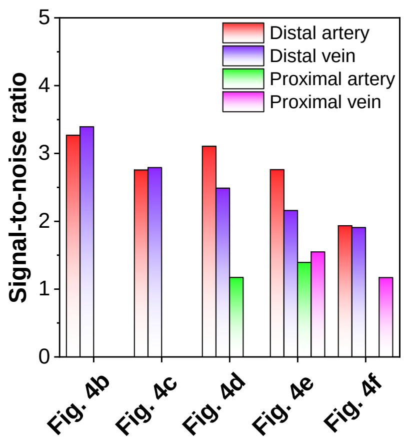

Supplementary Fig. 21. The signal-to-noise ratio of renal vessel (distal artery, distal vein, proximal artery, and proximal vein) of rabbits in different vascular anastomotic abnormalities corresponded to the end stage in Fig. 4.

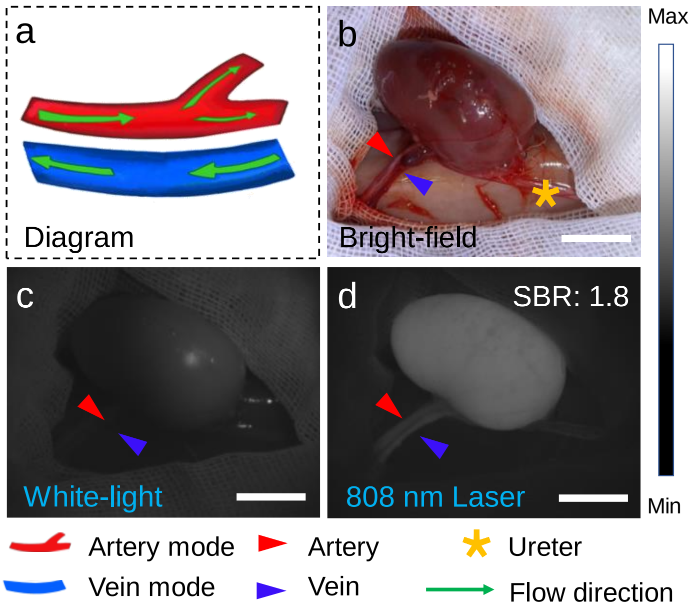

Supplementary Fig. 22. a-b The diagram (**a**) and bright-field image (**b**) of kidney region of rabbit. **c-d** The fluorescence images of donor kidney vessels after intravenous injection of ICG (300 μM, 2 mL) under laparoscopic-light (**c**) and 808 nm laser (**d**) irradiation with the same optical power density (20 mW cm^{-2}). Scale bar: 1 cm.

Supplementary Table 1. Photophysical characterization of previously reported organic NIR-II fluorophores[3-21].

Name	Excitation light	ε_{max} (L mol^{-1} cm^{-1})	λ_{em} (nm)	QY[a] (%)	Brightness (QY × ε)	Ref.
**Y6CT-NPs**	**White-light**	**8.26×10^4**	**947, 1030**	**16.12 8.62 (NIR-II)**	**13315.1 7120.1 (NIR-II)**	**This work**
FY6-NPs	808 nm laser	7.64×10^4	947, 1052	4.20	3208.8	3
BNDI-Me NPs	808 nm laser	16.4×10^4	1104	1.40	2296.0	4
CPTIC NFs	808 nm laser	14.5×10^4	1110	3.90	5655.0	5
BMIC-BO-4Cl NPs	880 nm laser	9.30×10^4	1010	2.29	2129.7	6
IR-FFCHP NPs	808 nm laser	~ 1.25×10^4	1038	7.30	912.5	7
L1013 NPs	808 nm laser	1.39×10^4	1013	9.9	1376.1	8
TT3-oCB NPs	793 nm laser	2.07×10^4	1062	4.60	952.2	9
TPE-BBT NPs	808 nm laser	1.40×10^4 (DMSO)	955	31.5	4410	10
TTQiT NPs	808 nm laser	3.89×10^4	1102	3.70	1439.3	11
TA1 NPs	808 nm laser	2.14×10^4	893	0.08 [b]	17.12	12
HL3 dots	808 nm laser	0.93×10^4	1125	11.70	1088.1	13
FD-1080 J-aggregate	1064 nm laser	5.00×10^4	1370	0.54	270	14
ICG	808 nm laser	12.0×10^4	822	1	1200	15
5H5	1064 nm laser	3.42×10^4 (MeCN)	1069	2.6	889.2	16
CX-3	808 nm laser	5.13×10^4 (DMSO)	1135	0.82	420.7	17
TPA-Et	808 nm laser	5.31×10^4 (CH_2Cl_2)	935	0.04 [c]	21.24	18
MB	623 nm LED	7.12×10^4	ND	0.20	142.4	19
BAF4 NPs	1064 nm laser	~ 0.75×10^4	ND	ND	ND	20
CCNU-1060 NPs	808 nm laser	1.60×10^4	1065	0.3	48	21

(a) The QY was re-calculated using QY of IR26 = 0.5% in DCE as a standard. (b) ICG (NIR-II QY = 1%) in water were used as the reference for calculating the fluorescence QY of TA1 NPs. (c) Absolute QY. These relevant molecular structures in the literature are as follows:

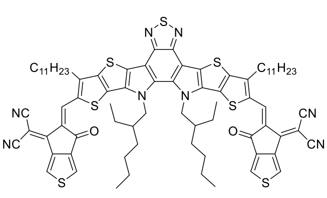
Y6CT-NPs

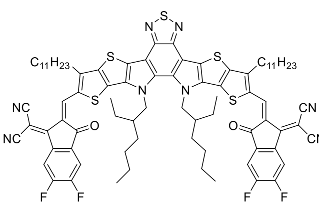
FY6-NPs

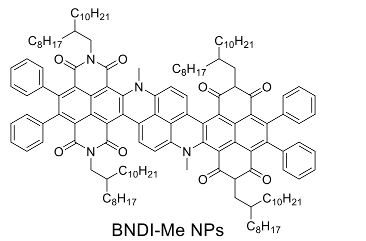
BNDI-Me NPs

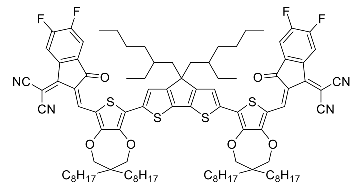
CPTIC NFs

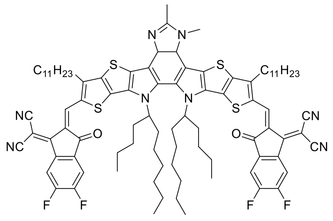
BMIC-BO-4Cl NPs

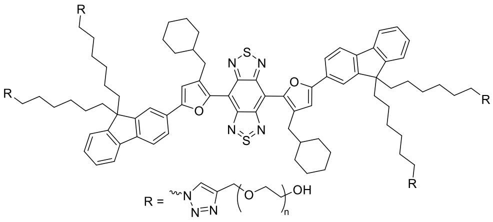
IR-FFCHP NPs

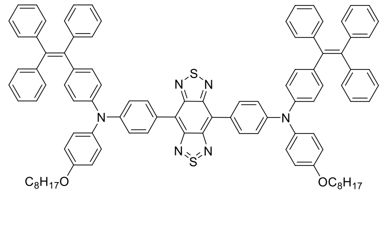
L1013 NPs

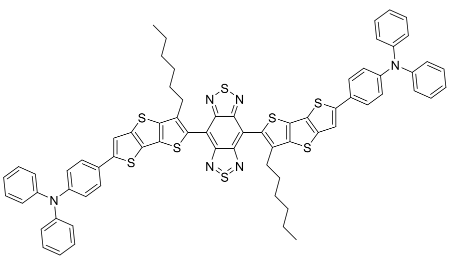
TT3-oCB NPs

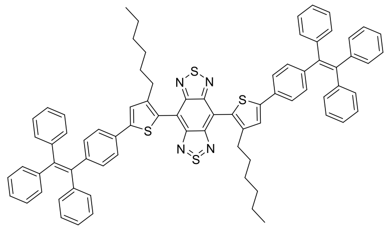
TPE-BBT NPs

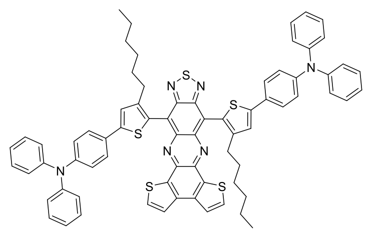
TTQiT NPs

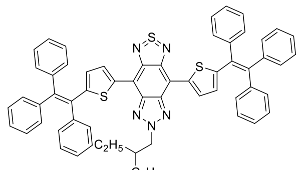
TA1 NPs

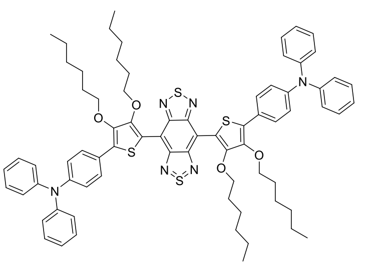
HL3 dots

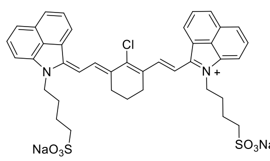
FD-1080

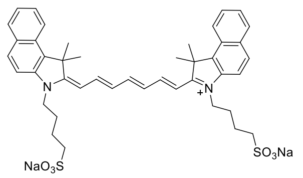
ICG

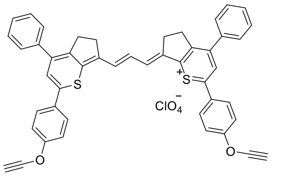
5H5

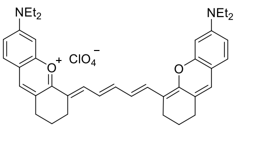
CX-3

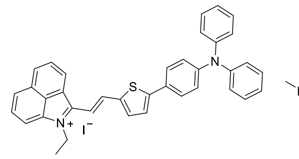
TPA-Et

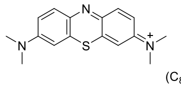
MB

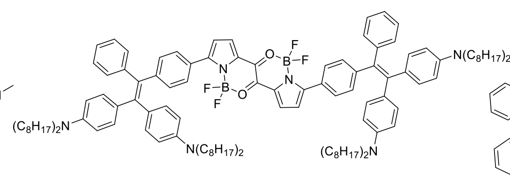
BAF4 NPs

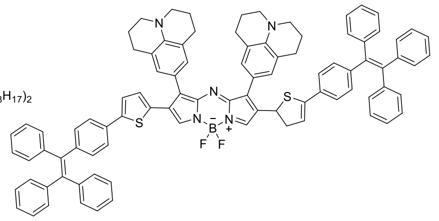
CCNU-1060 NPs

Supplementary Table 2. Crystallographic and structural refinement data of Y6CT.

Name	Y6CT
Empirical formula	$C_{78}H_{86}N_8O_2S_7$
Formula weight	1391.96
Temperature (K)	193.00
Crystal system	triclinic
Space group	P-1
a (Å)	16.5063(6)
b (Å)	16.8695(6)
c (Å)	17.9534(6)
α (°)	101.651(2)
β (°)	103.813(2)
γ (°)	1117.761(2)
Volume (Å^3)	4000.4(3)
Z	2
ρ_{calc}g/cm^3	1.156
μ/mm^{-1}	1.441
F (000)	1476.0
Crystal size/mm^3	0.13 × 0.12 × 0.1
Radiation	GaKα (λ = 1.34139)
2Θ range for data collection/°	5.386 to 107.814
Index ranges	-19 ≤ h ≤ 19, -20 ≤ k ≤ 20, -21 ≤ l ≤ 21
Reflections collected	44729
Independent reflections	14591 [R_{int} = 0.0642, R_{sigma} = 0.0780]
Data/restraints/parameters	14591/785/860
Goodness-of-fit on F^2	1.225
Final R indexes [I >= 2σ (I)]	R_1 = 0.1351, wR_2 = 0.3135
Final R indexes [all data]	R_1 = 0.2072, wR_2 = 0.3412
Largest diff. peak/hole / e Å^{-3}	1.48/-1.09
CCDC Number	2302102

Supplementary Table 3. Routine blood indexes of mice after different treatments (data were presented as mean ± SD derived from n = 3 independent samples per group).

	Saline	Y6CT-NPs
WBC (10^9 /L)	4.9 ± 0.46	4.6 ± 0.26
Lymph# (10^9 /L)	3.7 ± 0.26	3.4 ± 0.26
Mon# (10^9 /L)	0.1 ± 0.00	0.1 ± 0.00
Gran (10^9 /L)	1.1 ± 0.25	1.1 ± 0.10
Lymph% (%)	75.5 ± 3.45	73.9 ± 2.41
Mon% (%)	2.0 ± 0.21	2.2 ± 0.10
Gran# (%)	22.5 ± 3.87	23.9 ± 2.40
RBC (10^{12} /L)	8.75 ± 2.89	10.07 ± 2.16
HGB (g/L)	138 ± 27.71	158 ± 4.36
HCT (%)	40.4 ± 9.41	46.4 ± 5.30
MCV (fL)	46.2 ± 6.41	46.1 ± 6.68
MCH (pg)	15.7 ± 3.86	15.6 ± 0.53
MCHC (g/L)	341 ± 36.17	340 ± 56.47
RDW (%)	11.9 ± 3.06	14.1 ± 1.13
PLT (10^9/L)	809 ± 126.48	806 ± 109.78
MPV (fL)	5.1 ± 2.09	5.0 ± 1.49
PDW (%)	16.7 ± 0.30	16.2 ± 1.21
PCT (%)	0.412 ± 0.11	0.403 ± 0.13

Supplementary Table 4. Blood biochemistry test regarding liver and kidney function of the mice after different treatments (data were presented as mean ± SD derived from n = 3 independent samples per group).

	ALT (U/L)	AST (U/L)	ALB (g/L)	BUN (mmol/L)	CREA (μmol/L)	UA (μmol/L)
Saline	24.054 ± 3.61	123.376 ± 3.11	39.856 ± 5.00	12.210 ± 2.11	7.721 ± 2.87	17.322 ± 4.78
Y6CT-NPs	24.869 ± 2.97	121.420 ± 16.19	38.975 ± 2.85	11.879 ± 4.32	7.383 ± 0.70	14.837 ± 3.12

Supplementary Table 5. Routine blood indexes of the rabbit after different treatments (data were presented as mean ± SD derived from n = 3 independent samples per group).

	Saline	Y6CT-NPs
WBC (10^9/L)	5.0 ± 0.53	4.9 ± 0.50
Lymph# (10^9/L)	1.4 ± 0.23	1.2 ± 0.10
Mon# (10^9/L)	0.2 ± 0.10	0.2 ± 0.17
Gran (10^9/L)	3.4 ± 0.21	3.5 ± 0.40
Lymph% (%)	27.3 ± 1.84	23.9 ± 4.97
Mon% (%)	4.7 ± 0.90	5.2 ± 0.75
Gran# (%)	68.0 ± 1.76	70.9 ± 5.76
RBC (10^{12}/L)	5.12 ± 0.24	4.93 ± 0.48
HGB (g/L)	107 ± 8.19	102 ± 18.33
HCT (%)	32.5 ± 2.11	31.5 ± 1.84
MCV (fL)	63.5 ± 4.01	64 ± 9.53
MCH (pg)	20.8 ± 1.31	20.6 ± 1.10
MCHC (g/L)	329 ± 20.42	323 ± 25.23
RDW (%)	18.2 ± 1.15	17.7 ± 1.65
PLT (10^9/L)	821 ± 50.92	855 ± 213.18
MPV (fL)	4.8 ± 0.26	4.8 ± 0.40
PDW (%)	16.1 ± 0.98	16.3 ± 2.06
PCT (%)	0.394 ± 0.05	0.410 ± 0.13

Supplementary Table 6. Blood biochemistry test regarding liver and kidney function of the rabbit after different treatments (data were presented as mean ± SD derived from n = 3 independent samples per group).

	ALT (U/L)	AST (U/L)	ALB (g/L)	BUN (mmol/L)	CREA (μmol/L)	UA (μmol/L)
Saline	45.177 ± 3.61	43.889 ± 2.47	38.877 ± 3.84	19.426 ± 2.36	89.483 ± 7.11	3.642 ± 0.58
Y6CT-NPs	44.805 ± 1.67	41.179 ± 2.24	38.642 ± 1.68	19.113 ± 2.30	88.847 ± 7.55	3.187 ± 0.32

Supplementary References

1. Williams, A. T. R., Winfield, S. A. & Miller, J. N. Relative fluorescence quantum yields using a computer-controlled luminescence spectrometer. *Analyst* **108**, 1067-1071 (1983).
2. Hong, G. *et al*. Ultrafast fluorescence imaging in vivo with conjugated polymer fluorophores in the second near-infrared window. *Nat. Commun.* **5**, 4206 (2014).

3. Li, C. *et al.* Fluorination enhances NIR-II emission and photothermal conversion efficiency of phototheranostic agents for imaging-guided cancer therapy. *Adv. Mater.* **35**, e2208229 (2023).
4. Li, Y. *et al*. Incorporation of robust NIR-II fluorescence brightness and photothermal performance in a single large π-conjugated molecule for phototheranostics. *Adv. Sci.* **10**, e2204695 (2023).
5. Zhu, X. *et al*. High brightness NIR-II nanofluorophores based on fused-ring acceptor molecules. *Nano Research* **13**, 2570-2575 (2020).
6. Gu, Y. *et al*. Chlorination-mediated π-π stacking enhances the photodynamic properties of a NIR-II emitting photosensitizer with extended conjugation. *Angew. Chem. Int. Ed.* **62**, e202303476 (2023).
7. Liu, C. *et al.* Furan donor for NIR-II molecular fluorophores with enhanced bioimaging performance. *Research* **6**, 0039 (2023).
8. Wu, W. *et al*. An organic NIR-II nanofluorophore with aggregation-induced emission characteristics for *in vivo* fluorescence imaging. *Int. J. Nanomedicine* **14**, 3571-3582 (2019).
9. Shen, H. *et al.* Rational design of NIR-II AIEgens with ultrahigh quantum yields for photo- and chemiluminescence imaging. *J. Am. Chem. Soc.* **144**, 15391-15402 (2022).
10. Liu, S. *et al.* Incorporation of planar blocks into twisted skeletons: Boosting brightness of fluorophores for bioimaging beyond 1500 nanometer. *ACS Nano* **14**, 14228-14239 (2020).
11. Li, Y. *et al*. Promoted NIR-II fluorescence by heteroatom-inserted rigid-planar cores for monitoring cell therapy of acute lung injury. *Small* **18**, e2105362 (2022).
12. Guo, B. *et al*. Organic small molecule based photothermal agents with molecular rotors for malignant breast cancer therapy. *Adv. Funct. Mater.* **30**, 1907093 (2019).
13. Li, Y. *et al*. Novel NIR-II organic fluorophores for bioimaging beyond 1550 nm. *Chem. Sci.* **11**, 2621-2626 (2020).
14. Sun, C. *et al*. J-aggregates of cyanine dye for NIR-II *in vivo* dynamic vascular imaging beyond 1500 nm. *J. Am. Chem. Soc.* **141**, 19221-19225 (2019).
15. Zhou, H. J. & Ren, T. B. Recent progress of cyanine fluorophores for NIR-II sensing and imaging. *Chem. Asian J.* **17**, e202200147 (2022).
16. Ding, B. *et al*. Polymethine thiopyrylium fluorophores with absorption beyond 1000 nm for biological imaging in the second near-infrared subwindow. *J. Med. Chem.* **62**, 2049-2059 (2019).
17. Lei, Z. *et al*. Stable, wavelength-tunable fluorescent dyes in the NIR-II region for in vivo high-contrast bioimaging and multiplexed biosensing. *Angew. Chem. Int. Ed.* **58**, 8166-8171 (2019).

18. Teng, C. *et al*. Synthesis of strong electron donating-accepting type organic fluorophore and its polypeptide nanoparticles for NIR-II phototheranostics. *Nanomedicine* **44**, 102574 (2022).

19. Xue, D. *et al*. Structural and functional NIR-II fluorescence bioimaging in urinary system via clinically approved dye methylene blue. *Engineering* **22**, 149-158 (2023).

20. Jiang, Z. *et al*. A borondifluoride-complex-based photothermal agent with an 80 % photothermal conversion efficiency for photothermal therapy in the NIR-II window. *Angew. Chem. Int. Ed.* **60**, 22376-22384 (2021).

21. Huang, W. *et al*. Rigidity bridging flexibility to harmonize three excited-state deactivation pathways for NIR-II-fluorescent-imaging-guided phototherapy. *Adv. Healthc. Mater.* **10**, e2101003 (2021).
