# Supplementary material for: White-light activatable organic NIR-II luminescence nanomaterials for imaging-guided surgery
Source: Nat Commun. 2024 Jul 11;15:5832. doi: 10.1038/s41467-024-50202-6 (PMC11239823; doi:10.1038/s41467-024-50202-6)
Supplement: Supplementary file 2 — Reporting Summary [file 41467_2024_50202_MOESM2_ESM.pdf]

nature portfolio

Corresponding author(s): Jianguo Wang, Limin Wu

Last updated by author(s): Jun 25, 2024

Reporting Summary

Nature Portfolio wishes to improve the reproducibility of the work that we publish. This form provides structure for consistency and transparency in reporting. For further information on Nature Portfolio policies, see our Editorial Policies and the Editorial Policy Checklist.

Statistics

For all statistical analyses, confirm that the following items are present in the figure legend, table legend, main text, or Methods section.

n/a	Confirmed	
☐	☒	The exact sample size (*n*) for each experimental group/condition, given as a discrete number and unit of measurement
☐	☒	A statement on whether measurements were taken from distinct samples or whether the same sample was measured repeatedly
☐	☒	The statistical test(s) used AND whether they are one- or two-sided<br>*Only common tests should be described solely by name; describe more complex techniques in the Methods section.*
☐	☒	A description of all covariates tested
☒	☐	A description of any assumptions or corrections, such as tests of normality and adjustment for multiple comparisons
☐	☒	A full description of the statistical parameters including central tendency (e.g. means) or other basic estimates (e.g. regression coefficient) AND variation (e.g. standard deviation) or associated estimates of uncertainty (e.g. confidence intervals)
☐	☒	For null hypothesis testing, the test statistic (e.g. *F*, *t*, *r*) with confidence intervals, effect sizes, degrees of freedom and *P* value noted<br>*Give P values as exact values whenever suitable.*
☒	☐	For Bayesian analysis, information on the choice of priors and Markov chain Monte Carlo settings
☒	☐	For hierarchical and complex designs, identification of the appropriate level for tests and full reporting of outcomes
☒	☐	Estimates of effect sizes (e.g. Cohen's *d*, Pearson's *r*), indicating how they were calculated

Our web collection on statistics for biologists contains articles on many of the points above.

Software and code

Policy information about availability of computer code

Data collection	Structural characterization. The organic compounds synthesized were characterized for their structures and purities using 1H and 13C NMR spectroscopy with Bruker ARX 600 and ARX 500 spectrometers, using chloroform-d as the solvent and tetramethylsilane (TMS) as a reference. High-resolution mass spectra (HRMS) were obtained using a GCT Premier CAB 048 mass spectrometer to determine exact molecular weights. Molecular configurations were elucidated through single crystal X-ray diffractometer using Bruker smart Apex 2 and Bruker D8 Venture. Nanoparticle characterization: Particle size and morphology were examined using a Hitachi HT 7800 transmission electron microscope, with size distribution analyzed via dynamic light scattering (DLS) using an Omni NanoBrook device. Photophysical characterization: The UV absorption and photoluminescence (PL) spectra were recorded using a Shimadzu UV-2600i spectrophotometer and an FS5 Spectrofluorometer, respectively. Temperature curves were monitored using an FLIR E8-XT camera (FLIR System). Cellular experiment: The absorbance of each sample was measured using a microplate reader (BioTek) for MTT assay. NIR-II imaging: A Series III 900/1700 In Vivo Imaging System (Suzhou NIR-Optics Co., Ltd., China) was used for image acquisition. In addition, excitation sources for photophysical tests, cellular, and in vivo experiments included laparoscopic-light (XD-303-80W, LED cold light source) and an 808 nm laser (CNI laser, MDL-XD-808-5W).
Data analysis	Weak interaction analysis from single crystal structure were performed using Multiwfn 3.8(dev), and corresponding structure and IGM isosurfaces were generated using the VMD 1.9.3 program,Statistical analysis of data was conducted using SPSS 23 software.

For manuscripts utilizing custom algorithms or software that are central to the research but not yet described in published literature, software must be made available to editors and reviewers. We strongly encourage code deposition in a community repository (e.g. GitHub). See the Nature Portfolio guidelines for submitting code & software for further information.

Data

Policy information about availability of data

All manuscripts must include a data availability statement. This statement should provide the following information, where applicable:

- Accession codes, unique identifiers, or web links for publicly available datasets
- A description of any restrictions on data availability
- For clinical datasets or third party data, please ensure that the statement adheres to our policy

All original data generated in this study are provided in the Supplementary Information/Source Data file. The X-ray crystallographic coordinates for structures reported in this study have been deposited at the Cambridge Crystallographic Data Centre (CCDC), under deposition number 2302102. These data can be obtained free of charge from The Cambridge Crystallographic Data Centre via www.ccdc.cam.ac.uk/data_request/cif. There are no restrictions on data availability in the current work. Besides, the full image dataset is available from the corresponding author upon request.

Research involving human participants, their data, or biological material

Policy information about studies with human participants or human data. See also policy information about sex, gender (identity/presentation), and sexual orientation and race, ethnicity and racism.

Reporting on sex and gender	*Use the terms sex (biological attribute) and gender (shaped by social and cultural circumstances) carefully in order to avoid confusing both terms. Indicate if findings apply to only one sex or gender; describe whether sex and gender were considered in study design; whether sex and/or gender was determined based on self-reporting or assigned and methods used. Provide in the source data disaggregated sex and gender data, where this information has been collected, and if consent has been obtained for sharing of individual-level data; provide overall numbers in this Reporting Summary. Please state if this information has not been collected. Report sex- and gender-based analyses where performed, justify reasons for lack of sex- and gender-based analysis.*
Reporting on race, ethnicity, or other socially relevant groupings	*Please specify the socially constructed or socially relevant categorization variable(s) used in your manuscript and explain why they were used. Please note that such variables should not be used as proxies for other socially constructed/relevant variables (for example, race or ethnicity should not be used as a proxy for socioeconomic status). Provide clear definitions of the relevant terms used, how they were provided (by the participants/respondents, the researchers, or third parties), and the method(s) used to classify people into the different categories (e.g. self-report, census or administrative data, social media data, etc.) Please provide details about how you controlled for confounding variables in your analyses.*
Population characteristics	*Describe the covariate-relevant population characteristics of the human research participants (e.g. age, genotypic information, past and current diagnosis and treatment categories). If you filled out the behavioural & social sciences study design questions and have nothing to add here, write "See above."*
Recruitment	*Describe how participants were recruited. Outline any potential self-selection bias or other biases that may be present and how these are likely to impact results.*
Ethics oversight	*Identify the organization(s) that approved the study protocol.*

Note that full information on the approval of the study protocol must also be provided in the manuscript.

Field-specific reporting

Please select the one below that is the best fit for your research. If you are not sure, read the appropriate sections before making your selection.

☒ Life sciences ☐ Behavioural & social sciences ☐ Ecological, evolutionary & environmental sciences

For a reference copy of the document with all sections, see nature.com/documents/nr-reporting-summary-flat.pdf

Life sciences study design

All studies must disclose on these points even when the disclosure is negative.

Sample size	In the section of characterization of photophysical properties, cellular experiments and in vivo imaging, the values reported are expressed as mean standard deviation (SD). All statistical graphs, absorption spectrum, and fluorescent spectra were analyzed with OriginLab 2018. Each experiment included at least three replicates. For photostability in Fig. 1h, the statistical significance of differences between groups was performed by using an unpaired two-sided t-test method on the SPSS 23 software. A value of $P < 0.05$ was considered significant and were indicated with asterisks: $*P < 0.05$, $**P < 0.01$ and $***P < 0.001$.
Data exclusions	No data were excluded from the analyses.
Replication	All the synthesis, characterization, photophysical, and biological measurements are reproducible.
Randomization	Samples were allocated into experimental groups randomly.

Blinding	The investigators were blinded to group allocation during data collection and/or analysis.

Reporting for specific materials, systems and methods

We require information from authors about some types of materials, experimental systems and methods used in many studies. Here, indicate whether each material, system or method listed is relevant to your study. If you are not sure if a list item applies to your research, read the appropriate section before selecting a response.

Materials & experimental systems

n/a	Involved in the study	
☒	☐	Antibodies
☐	☒	Eukaryotic cell lines
☒	☐	Palaeontology and archaeology
☐	☒	Animals and other organisms
☒	☐	Clinical data
☒	☐	Dual use research of concern
☒	☐	Plants

Methods

n/a	Involved in the study	
☒	☐	ChIP-seq
☒	☐	Flow cytometry
☒	☐	MRI-based neuroimaging

Eukaryotic cell lines

Policy information about cell lines and Sex and Gender in Research

Cell line source(s)	The LO2 and NIH-3T3 cells lines used herein is purchased form ATCC cell banks.
Authentication	LO2 cell lines herein have been tested by ATCC and have a serial number of ATCC® CRL-12461. NIH-3T3 cells lines herein have been tested by ATCC and have a serial number of ATCC® CRL-1658.
Mycoplasma contamination	We confirm that LO2 and NIH-3T3 cells tested negative for mycoplasma contamination.
Commonly misidentified lines (See ICLAC register)	There are no misidentified cell lines in our study.

Animals and other research organisms

Policy information about studies involving animals; ARRIVE guidelines recommended for reporting animal research, and Sex and Gender in Research

Laboratory animals	Female BALB/c nude mice (6 weeks old and weighing 18-20 g) were purchased from Spiff Biotechnology Co., Ltd. (Beijing, China); The male/female New Zealand rabbits (3 months old and weighing 1.5~2.0 kg) were obtained from National Institutes for Food and Drug Control (Beijing, China).
Wild animals	*Provide details on animals observed in or captured in the field; report species and age where possible. Describe how animals were caught and transported and what happened to captive animals after the study (if killed, explain why and describe method; if released, say where and when) OR state that the study did not involve wild animals.*
Reporting on sex	Sex was not considered in study design.
Field-collected samples	*For laboratory work with field-collected samples, describe all relevant parameters such as housing, maintenance, temperature, photoperiod and end-of-experiment protocol OR state that the study did not involve samples collected from the field.*
Ethics oversight	All animals were cared for in accordance with the guidelines outlined in the Guide for the Care and Use of Laboratory Animals. The procedures were approved by the Institutional Animal Care and Use Committee at the Inner Mongolia University (IMU-2022-mouse-047 and IMU-2023-rabbit-048).

Note that full information on the approval of the study protocol must also be provided in the manuscript.

Plants

Seed stocks	*Report on the source of all seed stocks or other plant material used. If applicable, state the seed stock centre and catalogue number. If plant specimens were collected from the field, describe the collection location, date and sampling procedures.*
Novel plant genotypes	*Describe the methods by which all novel plant genotypes were produced. This includes those generated by transgenic approaches, gene editing, chemical/radiation-based mutagenesis and hybridization. For transgenic lines, describe the transformation method, the number of independent lines analyzed and the generation upon which experiments were performed. For gene-edited lines, describe the editor used, the endogenous sequence targeted for editing, the targeting guide RNA sequence (if applicable) and how the editor was applied.*
Authentication	*Describe any authentication procedures for each seed stock used or novel genotype generated. Describe any experiments used to assess the effect of a mutation and, where applicable, how potential secondary effects (e.g. second site T-DNA insertions, mosiacism, off-target gene editing) were examined.*
