# Supplementary material for: White-light activatable organic NIR-II luminescence nanomaterials for imaging-guided surgery
Source: Nat Commun. 2024 Jul 11;15:5832. doi: 10.1038/s41467-024-50202-6 (PMC11239823; doi:10.1038/s41467-024-50202-6)
Supplement: Supplementary file 3 — Description of Additional Supplementary Files [file 41467_2024_50202_MOESM3_ESM.pdf]

Title: Supplementary Movie 1:
Description: NIR-II fluorescence imaging of the renal artery and vein of the donor kidney region in the rabbit after intravenous injection of Y6CT-NPs.

Title: Supplementary Movie 2:
Description: NIR-II fluorescence imaging of the renal vasculature in rabbits with a normal anastomosis during kidney transplantation afte
